# Broad-Spectrum Antimicrobial Activity and Low Cytotoxicity against Human Cells of a Peptide Derived from Bovine α_S1_-Casein

**DOI:** 10.3390/molecules23051220

**Published:** 2018-05-19

**Authors:** Juncai Hou, Zhijing Liu, Songsong Cao, Haimei Wang, Chenggang Jiang, Muhammad Altaf Hussain, Shiyue Pang

**Affiliations:** 1 Key Laboratory of Dairy Science, Northeast Agricultural University, College of Food Science, Harbin 150030, China; liuzhijing22@126.com (Z.L.); Caodasong@126.com (S.C.); Wanghaimei66@126.com (H.W.); draltafdawar@gmail.com (M.A.H.); pangshiyue33@163.com (S.P.); 2 Harbin Veterinary Research Institute, CAAS, Harbin 150001, China; jcg5168@163.com

**Keywords:** antibacterial peptides, antimicrobial activity, hemolytic activity, human cell, cytotoxicity mechanism

## Abstract

The primary objective of this study was to improve our understanding of the antimicrobial mechanism of protein-derived peptides and to provide evidence for protein-derived peptides as food bio-preservatives by examining the antimicrobial activities, low cytotoxicity, stabilities, and mechanism of Cp1 (LRLKKYKVPQL). In this study, the protein-derived peptide Cp1 was synthesized from bovine α_S1_-casein, and its potential use as a food biopreservative was indicated by the higher cell selectivity shown by 11-residue peptide towards bacterial cells than human RBCs. It also showed broad-spectrum antimicrobial activity, with minimum inhibitory concentrations (MICs) of 64–640 μM against both gram-positive and gram-negative bacteria. The peptide had low hemolytic activity (23.54%, 512 μM) as well as cytotoxicity. The results of fluorescence spectroscopy, flow cytometry, and electron microscopy experiments indicated that Cp1 exerted its activity by permeabilizing the microbial membrane and destroying cell membrane integrity. We found that Cp1 had broad-spectrum antimicrobial activity, low hemolytic activity, and cytotoxicity. The results also revealed that Cp1 could cause cell death by permeabilizing the cell membrane and disrupting membrane integrity. Overall, the findings presented in this study improve our understanding of the antimicrobial potency of Cp1 and provided evidence of the antimicrobial mechanisms of Cp1. The peptide Cp1 could have potential applications as a food biopreservative.

## 1. Introduction

The rapid emergence of antibiotic resistance and microbial contamination in recent years has become a crucial problem worldwide in food safety as well as in food industries [1,2,3]. Thus, the development of new classes of antimicrobial agents has become a primary focus of many researchers [4]. Antimicrobial peptides (AMPs), also called host defense peptides, are primordial constituents of the innate immune system found in eukaryotic organisms and indicate broad-spectrum antimicrobial activity against microorganisms like viruses, bacteria, parasites, and fungi [5,6,7]. Moreover, antimicrobial peptides have been isolated from a wide range of sources including animals, plants, and bacteria [8]. Most of the AMPs show common characteristics because of having a positive charge. For example, AMPs generally consist of 12–50 amino acids, with 2–9 cationic residues and up to 50% hydrophobic amino acids [9,10]. Additionally, AMPs have low molecular weights, ranging from approximately 1 to 5 kDa [11]; these features (net charge and size) are important properties of peptides that allow AMPs to bind to or insert themselves into the membranes of bacterial [12], causing damage to bacterial membranes that lead to cell death [2]. Therefore, AMPs are presently receiving significant attention as potential alternatives to conventional antibiotics [13,14]. However, some AMPs are also cytotoxic to mammalian cells, which may limit the direct use of these peptides as therapeutics [15].

In recent years, AMPs, including various protein-derived peptides, have been identified from a diverse range of food sources such as milk products, cereals, soybeans, and fish, owing to their low hemolysis and toxicity [16,17]. AMPs from bovine milk proteins have been studied widely, and many AMPs have been shown to be generated by hydrolysis of milk proteins, particularly, bovine casein [18]. The earliest milk-derived antibacterial peptides were obtained by enzymatic hydrolysis of αs1-casein by chymotrypsin to obtain antibacterial properties. Protein-derived peptides could have potential applications as food biopreservatives [19]. For example, the protein-derived peptide Cp1 (LRLKKYKVPQL) was obtained from the hydrolysis of bovine α_S1_-casein, which intercepted the 11-amino acid of αS1-casein and showed higher antibacterial activity, as reported by McCann et al. [20] and Tang et al. [21]. Cp1 may have applications as a potential antimicrobial food biopreservative owing to low molecular mass and high antimicrobial activity compared with that of other protein-derived peptides. Although the isolation and characterization of Cp1 have been previously reported [21], the hemolytic activity, low cytotoxicity against human cells, and the exact mechanism by which the Cp1 exerts its antimicrobial effects have not been described. Moreover, the general antimicrobial mechanisms of protein-derived peptides have not been elucidated.

Thus, in order to resolve the problem of antibiotic resistance, which is produced by multicellular organisms as a defense mechanism against antibiotics, AMPs appear to be excellent candidates. Therefore, the objective of this study was to evaluate and examine the antimicrobial activity, hemolytic activity, low cytotoxicity against human cells, sensitivity, and exact mechanisms of Cp1 in order to improve our understanding of the antimicrobial mechanisms of protein-derived peptides and provide evidence for protein-derived peptides as a food biopreservative. Moreover, this study focused on obtaining more evidence to support the application of α_S1_-casein antimicrobial peptides as a biopreservative food byproduct as well as in the food industry.

## 2. Results

### 2.1. Characterization and Structure Variability of the Peptides

The Cp1 peptide (amino acid sequence: LRLKKYKVPQL) was synthesized; this 11-residue peptide related to amino acid residues 99–109 of bovine α_s1_-casein [20]. Melittin was purified from bee venom and is commonly used as a control peptide [22,23]. Table 1 summarizes the characterization of Cp1 and melittin. The measured molecular weights and theoretical molecular weights of each peptide were similar, supporting the successful synthesis of Cp1 and melittin. The two peptides were cationic (net charges ≥ 3), and the hydrophobicity (H) values of Cp1 and melittin were calculated to be 0.345 and 0.511, respectively. The hydrophobic moment (μH) values of Cp1 and melittin were 0.096 and 0.394, respectively, indicating the degree of amphipathicity of the peptides, and the theoretical isoelectric points (pIs) of the peptides were 10.46 and 12.02, respectively.

**Figure 1:**
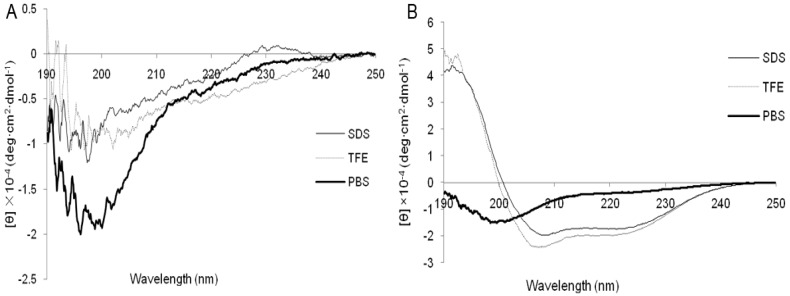
CD spectra of Cpl (**A**) and melittin (**B**). The peptides were dissolved in 10 mM PBS (pH 7.4), 50% TFE, and 30 mM SDS. Data are expressed as the mean residue ellipticities.

In different media the secondary structures of the peptides (10 mM sodium phosphate buffer [mimicking an aqueous environment], 50% TFE [mimicking the hydrophobic environment of the microbial membrane], and 30 mM SDS [mimicking the negatively charged prokaryotic membrane]) are shown in Figure 1. The CD spectra of Cp1 at 150 μM in different environments showed evidence of an unordered conformation with a negative ellipticity near 200 nm (Figure 1A). The CD spectra of melittin in 10 mM sodium phosphate buffer also showed evidence of an unordered conformation, with a population undergoing an α-helix conformation transition in TFE and SDS, as indicated by the minima of the spectra at 208 and 222 nm (Figure 1B).

### 2.2. Activities of the Peptides

#### 2.2.1. Antimicrobial Activity

Their bacteriostatic inhibitory concentrations, the antimicrobial activities of the two peptides were investigated, as indicated by the MICs (Table 2). The MICs of Cp1 against gram-negative and gram-positive bacteria ranged from 64 to 640 μM. In contrast, melittin exhibited strong antimicrobial activity beside all bacterial strains in our analysis, with MICs ranging from 1 to 8 μM.

#### 2.2.2. Hemolytic Activity

**Figure 2:**
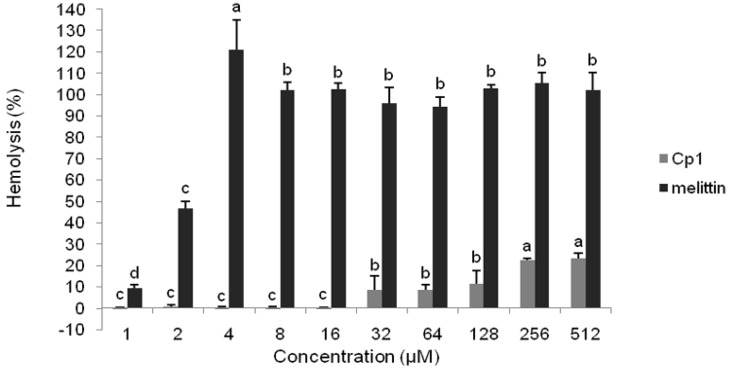
Hemolytic activities of Cp1 and melittin. Means in the same concentration with different superscript letters are significantly different (*p* < 0.05).

The hemolytic activities of the peptides are shown in Table 2 and Figure 2. The hemolytic activities of the peptides against human RBCs were tested at final peptide concentrations ranging from 1 to 512 μM. The Cp1 peptide showed no hemolytic activity at concentrations of up to 16 μM. The hemolytic activity of the peptide was only 23.54% at the highest concentration of 512 μM. The control peptide melittin had significantly higher hemolytic activity than Cp1, with a hemolysis rate of 46.80% at 2 μM (*p* < 0.05).

#### 2.2.3. Cytotoxicity Activity

**Figure 3:**
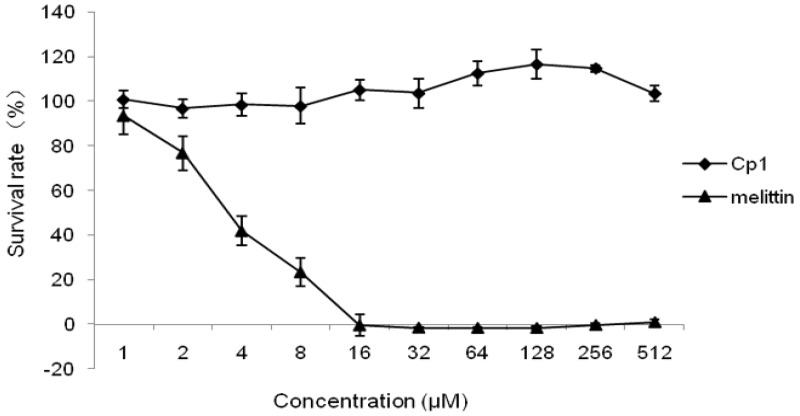
Cytotoxicity of Cp1 and melittin in 293 cells.

The two peptides were verified for cytotoxic activity in 293 cells (Figure 3). The Cp1 peptide had no cytotoxic activity at concentrations of 1–512 μM. In contrast, the control peptide melittin exhibited greater cytotoxic activity, with cell survival rates of 41.95%, 23.40%, and 0% at concentrations of 4, 8, and 16 μM, respectively.

#### 2.2.4. Salt Sensitivity

To check salt sensitivity, the antimicrobial activity of peptides against *E. coli* ATCC 25922 and *L. monocytogenes* CMCC 54004 was evaluated in the presence of 150 mM NaCl, 4.5 mM KCl, 6 μM NH_4_Cl, 1 mM MgCl_2_, 2 mM CaCl_2_, or 4 μM FeCl_3_ (Table 3). Monovalent (Na^+^, K^+^, and NH_4_^+^) and trivalent (Fe^3+^) cations did not affect the MIC values of Cp1 against *E. coli* ATCC 25922, whereas divalent (Mg^2+^ and Ca^2+^) cations increased the MIC values from 64 μM to 128 μM, with concurrent decreases in antimicrobial activity. Compared with its activity against gram-negative bacteria, the Cp1 peptide exhibited reduced antimicrobial activity against *L. monocytogenes* CMCC 54004 in the presence of monovalent (NH_4_^+^) and divalent (Ca^2+^) cations, particularly two-unit cations. Moreover, other cations, including monovalent (Na^+^ and K^+^), divalent (Mg^2+^), and trivalent (Fe^3+^) cations, did not affect the antimicrobial activities of the peptides. Additionally, at physiological concentrations, none of the cations affected the antimicrobial activity of melittin.

### 2.3. Mechanism of Action of the Peptides

#### 2.3.1. Evaluation of Outer Membrane Permeability

**Figure 4:**
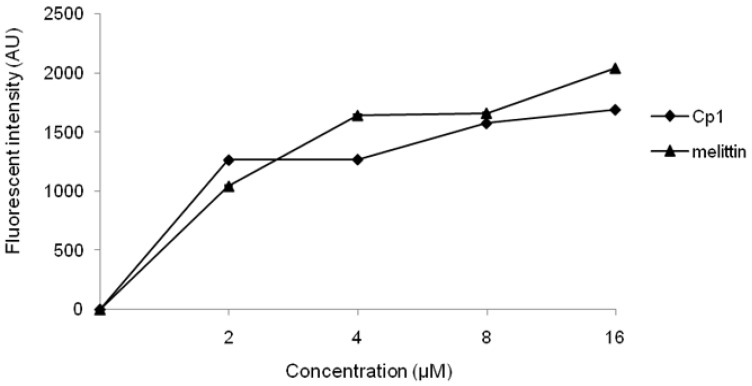
Outer membrane permeability of the peptides. The uptake of NPN by *E. coli* UB1005 in the presence of different concentrations of the peptides was determined using NPN assays. NPN uptake was monitored at an excitation wavelength of 350 nm and an emission.

The capability to permeabilize the bacterial outer membrane of peptides was assessed through NPN uptake assays. Normally, NPN is excluded from *E. coli* cells but it can traverse cells with a damaged outer membrane, increasing the fluorescence intensity. NPN uptake assays showed that the peptides permeabilized the outer membrane of *E. coli* cells (Figure 4). Compared with melittin, Cp1 had a higher capacity to permeabilize the outer membrane at 0–2 μM and had a poorer capacity to permeabilize the outer membrane at 4–16 μM. However, both peptides could permeabilize the outer membrane at all examined concentrations (0–16 μM).

#### 2.3.2. Evaluation of Inner Membrane Permeability

**Figure 5:**
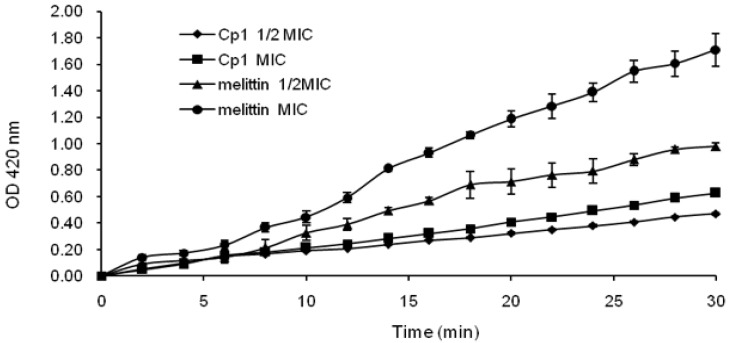
Inner membrane permeability of the peptides. Hydrolysis of ONPG due to release of cytoplasmic β-gaIactosidase in *E. coli* UB1005 cells treated with peptides at l × MIC and 1/2 MIC was measured spectroscopically at an absorbance of 420 nm as a function of time.

To further investigate the inner membrane permeability of the peptides, we used ONPG assays. As shown in Figure 5, Cp1 and melittin induced rapid increases in the permeability of the inner membrane at 1× MIC and 1/2 MIC, and both peptides maintained a steady increasing trend. The inner membrane permeability of the peptides at 1× MIC was higher than that at 1/2 MIC.

#### 2.3.3. Cytoplasmic Membrane Electrical Potential

**Figure 6:**
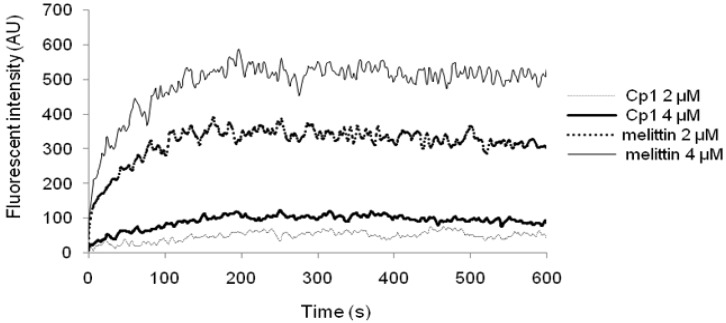
Cytoplasmic membrane depolarization of *E. coli* UB1005 treated with Cp1 and melittin at final concentrations of 2 or 4 μM.

In diSC_3_-5 assays, the fluorescence of the dye is quenched in the cytoplasmic membrane, and the membrane is dissipated and depolarized after the peptide added. The dye is then released into the medium, with a conspicuous increase in the fluorescence intensity. As shown in Figure 6, Cp1 and melittin had the ability to depolarize the cytoplasmic membrane of *E. coli* UB1005 cells in a concentration- and time-dependent manner. The results showed that melittin induced a more rapid increase in fluorescence intensity than Cp1 at 2 or 4 μM, whereas Cp1 was able to permeabilize the bacterial cell membrane, even at concentrations lower than the MIC (128 μM). These results suggested that the capacity to depolarize the cytoplasmic membrane was not needed for realizing strong antimicrobial activity.

#### 2.3.4. Flow Cytometry

**Figure 7:**
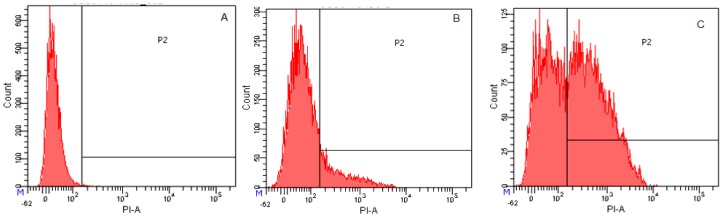
Flow cytometric analysis. Membrane damage in *E. coli* 25922 treated with the peptides was evaluated by measuring the fluorescent intensity of propidium iodide (PI) at 4 °C for 30 min. (**A**) No peptide, negative control; (**B**) Cp1 at 1 × MIC; (**C**) melittin at 1 × MIC.

To determine whether Cp1 and melittin permeabilized or otherwise affected the cell membrane, we used staining with the DNA intercalating dye PI, which stains nucleic acids in *E. coli* cells as a measure of membrane disruption and cell death. The results were analyzed by flow cytometry. As shown in Figure 7A, 99.7% of cells showed no PI fluorescence in the absence of the peptide, demonstrating that cell membranes were intact. Cp1 caused an increase in PI fluorescence with 17.6% cells stained at 1 × MIC (Figure 7B). Cells treated with melittin at its MIC showed a rapid increase in PI fluorescence, with 57.3% of cells stained (Figure 7C). These results indicated that Cp1 and melittin could induce membrane damage in bacterial cells.

#### 2.3.5. SEM

**Figure 8:**
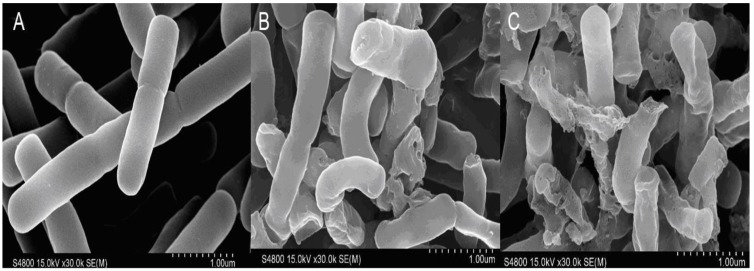
Scanning electron micrographs of *E. coli* 25922 treated with Cp1 and melittin. (**A**) Control, no peptides; (**B**) Cp1 at 1 × MIC for 2 h; (**C**) melittin at 1 × MIC for 2 h.

The morphological characteristics of the bacterial surface were further observed by SEM (Figure 8). From the SEM images, we found that untreated *E. coli* cells had an intact, smooth surface without any visible cell lysis or debris (Figure 8A), indicating that the bacterial cells were healthy. However, treatment with Cp1 or melittin for 2 h at the corresponding MC caused gross morphological changes in the bacteria, including partial blebbing and budding, extracellular debris, and irregular shapes (Figure 8B,C).

#### 2.3.6. TEM

**Figure 9:**
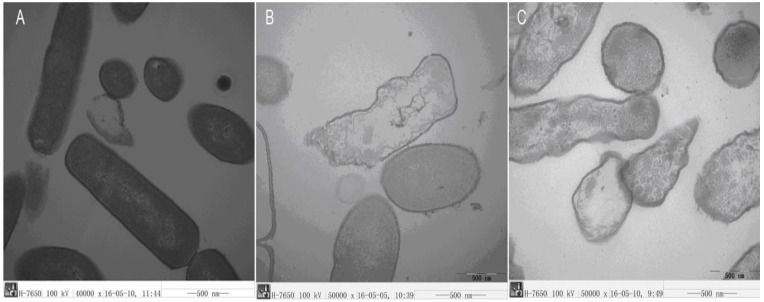
Transmission electron micrographs of *E. coli* 25922 treated with Cp1 and melittin. (**A**) Control, no peptides; (**B**) Cp1 at 1 × MIC for 2 h; (**C**) melittin at 1 × MIC for 2 h.

The ultrastructural features of *E. coli* cells were observed using Transmission Electron Microscopy TEM (Figure 9). The control *E. coli* cells presented a smooth, intact membrane surface and compact internal structure without peptides (Figure 9A). However, after treatment with Cp1 or melittin for 2 h, severe damage was observed, including pore formation and efflux of content (Figure 9B,C). In addition, major rupture of cell membranes and leakage of intracellular contents were clearly observed (Figure 9B), and dissolution of the cytoplasmic space and clumping or coagulation of cytoplasm were observed (Figure 9C).

## 3. Discussion

Adoption of secondary structure in the membrane-mimicking milieu is thought to account for the antimicrobial activity and cytotoxicity of AMPs. In this study, we found that the secondary structure of Cp1 in different environments showed an unordered conformation, indicating that the mechanism through which Cp1 disrupted cell membranes was random coil formation. Moreover, although melittin in sodium phosphate buffer exhibited an unordered conformation, it showed an α-helix structure in the membrane-like environment of TFE and SDS solutions, confirming that the structure of melittin disrupted cell membranes through formation of α-helix structures as the buffer changed from sodium phosphate buffer to TFE and SDS, similar to the results described by Pandey et al. [24] and Asthana et al. [25]. Ma et al. [26] reported that α-helical AMPs have higher antimicrobial activity, which may explain why the antimicrobial activity of Cp1 was lower than that of melittin. However, the helicity of AMPs is associated with hemolytic activity [27,28], which may be why the hemolytic activity of Cp1 was lower than that of melittin. Oren et al. [29] also found that melittin diastereomers lost their α-helical structure, abrogating their hemolytic activity toward human RBCs.

As reported in previous studies, most naturally occurring AMPs are cationic [30]. Cationic AMPs have a net positive charge due to the absence of acidic residues (glutamate or aspartate) and excess of cationic (arginine, lysine, or histidine) residues and the presence of approximately 30–50% hydrophobic residues [31]. The bacterial membrane contains zwitterionic phospholipids and carries a slight negative charge, which would attract cationic AMPs, allowing them to insert into membranes at the membrane interface, leading to increased membrane permeability and subsequent cell death [32,33]. Cationic residues can increase electrostatic interactions between negatively charged surface of the bacterial membrane and peptides [34,35]; the increasing hydrophobicity of AMPs facilitates their insertion into the membrane lipid bilayer within a certain range [36,37]. In this study, Cp1 was characterized as a cationic AMP having four cationic (arginine and lysine) residues and five hydrophobic (leucine, valine, and proline) residues. As observed in antimicrobial assays, Cp1 had broad-spectrum antimicrobial activity against gram-positive and gram-negative bacteria in the range from 64 to 640 μM. McCann et al. [20] reported that Cp1 exhibits MICs ranging from 90 to greater than 720 μM against various gram-negative and gram-positive bacteria; these MICs were slightly lower than those in our current study, possibly because of differences in peptide purity and bacterial strains. Melittin exhibited higher antimicrobial activity against all bacterial strains, which is similar to reports by Asthana et al. [25] and Zhu et al. [38]. Although the antimicrobial activity of Cp1 was lower than that of melittin, Cp1 had higher antimicrobial activity than other protein-derived peptides, such as isracidin and VYQHQKAMKPWIQPKTKVIPYVRYL, originating from α_s1_-casein and α_s2_-casein, with MICs of 100–1000 and 332–4664 μg/mL, respectively [20,39].

Hemolysis and cytotoxicity are major parameters used to assess peptide toxicity in mammalian cells [37]. In this study, we found that the hemolytic activities and cytotoxicity of Cp1 were low, such that Cp1 had high cell selectivity. These results indicated that this peptide could be developed as a promising food biopreservative. Notably, the effects of cations such as Na^+^ and Mg^2+^ can hamper the electrostatic interactions between peptides and membranes of bacteria and may therefore affect AMP activity [40,41,42]. Many studies have reported that some cations decrease the antimicrobial activity of peptides. For example, Bellamy et al. [43] found that the antibacterial activity of lactoferricin B was decreased in the presence of Na^+^, K^+^, Mg^2+^, or Ca^2+^ ions. Human cathelicidin LL-37 and pleurocidin isolated from *Pleuronectes americanus* are also salt sensitive [44,45]. In this study, we found that Ca^2+^ exhibited a higher antagonistic effect on Cp1 against *E. coli* ATCC 25922 and *L. monocytogenes* CMCC 54004 at the physiological concentration. Mg^2+^ and NH_4_^+^ also inhibited the activity of Cp1 against *E. coli* ATCC 25922 and *L. monocytogenes* CMCC 54004, respectively. However, the results showed that salts did not affect the antimicrobial activity of melittin. A previous study demonstrated that the inhibition of antibacterial activity by Mg^2+^ was lower than that by Ca^2+^ [41], consistent with our current findings. However, we found that two monovalent cations (Na^+^ and K^+^) did not affect the antimicrobial activity of Cp1 when used at their physiological concentrations. This may be due to the net positive charge, which could slightly decrease the effects of Na^+^ on electrostatic interactions [46]. Moreover, divalent cations interfere with opposition for membrane binding between peptides and cations and can increase membrane rigidity [47,48]. Taken together, our findings suggested that Cp1 may have lower sensitivity to salts, making it particularly promising for use as a food biopreservative.

Previous studies have shown that the predominant antimicrobial mechanism of cationic AMPs lies in membrane destabilization or transmembrane pore formation [48]. Phosphatidylglycerol (PG), cardiolipin (CL), and phosphatidylserine (PS) are predominant components in the bacterial membrane, maintaining the negative net charge and promoting the binding of cationic AMPs [49,50]. Then, AMPs perturb the lipid bilayer through membrane permeabilization, possibly by surface insertion, followed by membrane thinning [51,52], and disrupt the bacterial cell membrane, causing outflow of cytoplasmic contents and ultimate cell death [37]. Membrane permeabilization is the most essential characteristic of peptide-membrane interactions and plays an important role in measuring the activity of cationic AMPs [38]. In this study, we also found that Cp1 and melittin had concentration-dependent effects on the permeabilization of the outer membrane of *E. coli* UB1005. Both Cp1 and melittin had the ability to permeabilize the inner membrane to ONPG at 1× MIC and 1/2 MIC, indicative of strong membrane-permeabilizing ability, with concentration-dependent effects. From flow cytometric analysis, we found that Cp1 and melittin killed bacteria by damaging cytoplasmic membrane integrity. Our SEM and TEM results further confirmed that Cp1 and melittin had potent interactions with the membrane structure, disrupted the cell membrane, and allowed the intracellular contents of the bacterial cells to leak out through the membrane. Taken together, these results demonstrated that Cp1 and melittin caused damage to the cytoplasmic membrane.

## 4. Materials and Methods

The peptide Cp1 and melittin were purchased and synthesized by GL Biochem (Shanghai, China) and identified via matrix-assisted laser desorption/ionization time-of-flight mass spectrometry (MALDI-TOF MS, Linear Scientific Inc., Duquesne, PA, USA), using a-cyano-4-hydroxycinnamic acid (HCCA) as the matrix. The purity of peptide was confirmed to be greater than 95% by analytical reversed-phase high-performance liquid chromatography (RP-HPLC) (LC 3000, Beijing, China). By using electrospray ionization mass spectrometry (ESI-MS) analysis, tentative molecular masses of the peptides were assessed.

Essential sequences, mean hydrophobicity, hydrophobic moment values, and helical wheel projections were evaluated using the methods of Zhu et al. [38]. Before assessments, peptide was liquefied in deionized water at a concentration of 2.56 mM.

*E.coli* ATCC 25922, *E. coli* UB1005, *S. pullorum* C7913, *S. Typhimurium* CMCC 50071, *Staphylococcus aureus* ATCC 29213, and *Listeria monocytogenes* CMCC 54004 were obtained from Harbin Veterinary Research Institute, CAAS (Harbin, China). From healthy blood donors red blood cells were extracted. The human embryonic kidney epithelial 293 cells used in this study were also obtained from Harbin Veterinary Research Institute, CAAS.

Mueller–Hilton broth (MHB) and Mueller–Hilton Agar (MHA) powder were bought from AoBoX (Beijing, China). Sodium dodecyl sulfate (SDS), trifluoroethyl alcohol (TFE), Triton X-100, o-nitrophenyl-b-d-galactopyranoside (ONPG), 3,30-dipropylthiadicarbocyanine (diSC3-5), HEPES, *N*-phenyl-1-napthylamine (NPN), propidium iodide (PI) were obtained from Sigma-Aldrich (Shanghai, China). NaCl, KCl, NH_4_Cl, MgCl_2_ and FeCl_3_ were all of analytical grade and obtained from Kermel (Tianjin, China).

### 4.1. Antimicrobial Assay

As described previously, by the CLSI broth microdilution method the MICs of the peptides were measured [53,54]. Concisely, the bacteria were diluted to a final concentration of 10^5^ CFU/mL in MHB. Two-fold serial dilutions of peptides in 12 serial 96-well plates were diluted in bovine serum albumin (BSA; 0.2% with 0.01% acetic acid). Fifty microliters of diluted bacteria were added to 50 μL BSA containing two-fold serial dilutions of peptides in each well. For a period of 18 h the plates were incubated at 37 °C. The minimum peptide concentration (MIC) was defined as the point at which no bacterial growth was observed.

### 4.2. Hemolytic Activity

For the measurement of hemolytic activity through the method of Stark et al., each peptide was measured [55]. Briefly, fresh human RBCs were obtained and stored at 4 °C. One milliliter of human RBCs was washed three times and resuspended in 10 PBS, and 50 μL of the human red blood cell solution were added to 50 μL PBS containing two-fold serial dilutions of peptides in each well. For 1 h the plates were kept in an incubator at 37 °C. By centrifugation and removal of supernatants, the discharge of hemoglobin was determined by calculating the absorbance using a microplate reader. As negative and positive controls, erythrocyte suspensions in PBS and 0.1% Triton X-100, were used separately.

### 4.3. Cytotoxicity

The cytotoxicity of each peptide was determined in 293 cells using MTT assays, as reported previously [56]. The preparation and storage methods for MTT and cell suspensions were previously described by Schmidtchen et al. [57]. Briefly, 293 cells were incubated overnight in 96-well plates with 10% fetal calf serum and DMEM at 37 °C in 5% CO_2_. By using DMEM two-fold serial dilutions of peptides were prepared. AMPs added on the plates and subjected to incubator for 20–24 h at 37 °C containing 5% CO_2_. Next, 40 μL MTT solution was added to every well, and plates were subjected to incubator for 4 h at 37 °C. Finally, 150 μL DMSO added to the wells, for 10 min the plates were shaken, and absorbance was measured by using a microplate reader at 492 nm.

### 4.4. Salt Sensitivity Assays

For checking salt sensitivity standard methods was adopted. Salts can affect the MIC values of peptides. Therefore, the salt sensitivities of the peptide was dignified using the way reported by Zhu et al. [38], with modifications. To inspect the effect of each salt on the antibacterial activities of the peptides different concentrations of physiological salts (150 mM NaCl, 4.5 mM KCl, 6 μM NH_4_Cl, 1 mM MgCl_2_, 2 mM CaCl_2_, and 4 μM FeCl_3_) were added to the incubation buffer. The same step was repeated for each peptide and findings were noted.

### 4.5. Circular Dichroism (CD) Measurements

To inspect the secondary structures of the peptides CD spectra were noted using a Jasco J-810 Spectropolarimeter with a 1-mm quartz cuvette. The peptides were dissolved in 10 mM PBS (pH 7.4), 50% TFE, and 30 mM SDS micelles. Spectra were monitored at standard temperature and speed, i.e., at 25 °C and noted in the range of 190 to 250 nm [11].

### 4.6. Outer Membrane Permeability Assay

The standard procedure was adopted for the assay, as given in the company booklet. The major steps were: By calculating integration of the fluorescent dye NPN into the outer membrane of *E. coli* UB1005, the ability of Cp1 and melittin to improve the outer membrane permeability of gram-negative bacteria was determined as described by Xu et al. [49] and Subbalakshmi et al. [58]. Briefly, the basic steps for assay were: cultures of *E. coli* UB1005 were diluted in MHB medium at 37 °C and grown to an OD_600_ of 0.2 in HEPES buffer overnight. The cell suspension was then mixed with 1 mM NPN, and the background fluorescence was noted at standard values of wavelengths until there was no additional increase in fluorescence.

### 4.7. Inner Membrane Permeability Assay

The standard procedure was adopted for the detection of permeability of inner membrane. The major steps were: By measuring β-galactosidase activity utilizing ONPG as a substrate, the permeability of the inner membrane of *E. coli* UB1005 by the peptides was determined [27,59]. *E. coli* UB1005 was cultured in MHB (containing 2% lactose) until reaching the logarithmic phase and obtained by centrifugation (5000× *g*, 5 min). The bacteria were diluted to an OD_600_ of 0.05 with 10 mM PBS (containing 1.5 mM ONPG). Different amounts of peptide (1× MIC and 1/2 MIC) were added and data were recorded every 2 min from 0 to 30 min at OD_420_.

### 4.8. Cytoplasmic Membrane Depolarization Assay

The steps for depolarization assay were performed according to standard procedure as given in company booklet. By using the dye diSC3-5, by modifying of the method of Friedrich et al., the depolarization activity of the cytoplasmic membrane of *E. coli* UB1005 by the peptides was noted [60], as described by Dong et al. [46]. *E. coli* UB1005 cells in the logarithmic phase were diluted with 5 mM HEPES (containing 20 mM glucose), and 0.4 mM diSC_3_-5 was added. KCl was then added to a concentration of 100 mM. Different amounts of peptide were added, and the fluorescence intensity was monitored from 0 to 600 s.

### 4.9. Flow Cytometry

For analysis of membrane integrity after peptide treatment, *E. coli* ATCC 25922 cells in the mid-log phase in MHB medium under continuous trembling at 200 rpm were resuspended in PBS (pH 7.2) to an OD_600_ of 0.2. The next step was mixing with peptides at 1 × MIC and incubated for 30 min at 37 ℃ with constant shaking (140 rpm). The control sample lacked the peptide. The bacterial cells were gained by centrifugation and then washed with PBS, subjected to an incubator with the bacterial suspension at a fixed PI concentration of 10 mg/mL for 30 min at 4 ℃ and then washed with superfluous PBS. Data were recorded using a FACScan instrument (Becton-Dickinson, San Jose, CA, USA) [61].

### 4.10. Scanning Electron Microscopy (SEM)

As described previously by Juba et al., SEM sample preparation was carried out [62], with modifications. *E. coli* ATCC 25922 cells in the mid-log phase in MHB medium under continuous shaking at 200 rpm were resuspended in PBS (pH 7.2) to an OD_600_ of 0.2. The bacterial cells were subjected to incubator at 37 °C under constant shaking at 200 rpm for 2 h with Cp1 or melittin at 1 × MIC. The control sample lacked the peptide. The bacterial cells were gained by centrifugation and washed with PBS, then fixed with 2.5% glutaraldehyde. Next, the samples were washed with PBS and dehydrated for 15 min in a graded ethanol (50%, 70%, 90%, and 100%). Finally, the bacterial specimens were observed by SEM after lyophilization and gold coating (Hitachi S-4800; Hitachi, Tokyo, Japan).

### 4.11. Transmission Electron Microscopy (TEM)

The standard procedure for TEM was adopted as described for SEM, TEM sample preparation was conducted [34,63]. *E. coli* ATCC 25922 cells were treated with 2.5% glutaraldehyde and washed with PBS. The samples were then postfixed with 2% osmium tetroxide for 2 h, washed with PBS, and dehydrated in graded ethanol (50%, 70%, 90%, and 100%). Finally, sections were performed with an ultramicrotome and thoroughly observed with a Hitachi H-7650 TEM.

### 4.12. Statistical Analysis

Values for peptide analysis were expressed as means ± standard deviations. By using one-way analysis of variance (ANOVA) in SPSS 17.0, the obtained data were analyzed statistically, with a significance level of 5%.

## 5. Conclusions

In this study, we found that Cp1 ensured broad-spectrum antimicrobial activity and low hemolytic and toxic effects. Moreover, Cp1 was active in the presence of physiological salts. The results also revealed that Cp1 could permeabilize the cell membrane and disturb membrane integrity, causing cell death. Overall, the findings presented in this study improve our understanding of the antimicrobial potency of Cp1 and provide insight into the antimicrobial mechanisms of Cp1. It is concluded that peptide Cp1 could have potential applications as a food biopreservative due to its valuable features like broad-spectrum activity and low hemolytic and toxic effects.

## Figures and Tables

**Table 1:** Characterization of each peptide.

Peptides	Sequence	Theoretical Mw (Da)	Measured Mw (Da)	Net Charge	H	μH	pI
Cp1	LRLKKYKVPQL-NH_2_	1385.76	1384.79	+4	0.345	0.096	10.46
Melittin	GIGAVLKVLTTGLPALISWIKRKRQQ-NH_2_	2847.49	2847.51	+5	0.511	0.394	12.02

The mean hydrophobicity and hydrophobic moment values were calculated online at http://heliquest.ipmc.cnrs.fr/. The theoretical pIs and theoretical molecular weights were calculated online at http://web.expasy.org/compute_pi/.

**Table 2:** Antimicrobial and hemolytic activities of the peptides.

Peptides	Minimum Inhibitory Concentration (MIC, μM) *	
	Cp1	Melittin
Gram-negative bacteria		
*E. coli* ATCC 25922	64	1
*E. coli* UB1005	128	2
*Salmonella pullorum* C7913	256	8
*Salmonella enterica subsp enterica* CMCC 50071	256	2
Gram-positive bacteria		
*Staphylococcus aureus* ATCC 29213	640	2
*L. monocytogenes* CMCC 54004	64	1
MHC ^†^	32	1

* The minimum inhibitory concentration (MIC) was determined as the lowest concentration of peptide that inhibited bacteria growth; ^†^ The MHC was the minimum hemolytic concentration that caused 5% hemolysis of human red blood cells.

**Table 3:** MIC values of peptides in the presence of physiological salts.

Peptides	Control	NaCl *	KCl *	NH_4_Cl *	MgCl_2_ *	CaCl_2_ *	FeCl_3_ *
Gram-negative strain *E. coli* 25922							
Cp1	64	64	64	64	128	128	64
Melittin	1	1	1	1	1	1	1
Gram-positive strain *L. monocytogenes* 54004							
Cp1	64	64	64	128	64	256	64
Melittin	1	1	1	1	1	1	1

* The final concentrations of NaCl, KCl, NH_4_Cl, MgCl_2_, CaCl_2_, and FeCl_3_ were 150 mM, 4.5 mM, 6 μM, 1 mM, 2 mM, and 4 μM, respectively, and the control MIC values were determined in the absence of these physiological salts.
